# Acidic Microenvironments Found in Cutaneous *Leishmania* Lesions Curtail NO-Dependent Antiparasitic Macrophage Activity

**DOI:** 10.3389/fimmu.2022.789366

**Published:** 2022-04-14

**Authors:** Linus Frick, Linda Hinterland, Kathrin Renner, Marion Vogl, Nathalie Babl, Simon Heckscher, Anna Weigert, Susanne Weiß, Joachim Gläsner, Raffaela Berger, Peter J. Oefner, Katja Dettmer, Marina Kreutz, Valentin Schatz, Jonathan Jantsch

**Affiliations:** ^1^ Institute of Clinical Microbiology and Hygiene, University Hospital of Regensburg and University of Regensburg, Regensburg, Germany; ^2^ Department of Internal Medicine III, University Hospital Regensburg, Regensburg, Germany; ^3^ Leibniz Institute for Immunotherapy, Regensburg, Germany; ^4^ Institute of Functional Genomics, University of Regensburg, Regensburg, Germany

**Keywords:** pH, *Leishmania*, macrophages, NO, NOS2

## Abstract

Local tissue acidosis affects anti-tumor immunity. In contrast, data on tissue pH levels in infected tissues and their impact on antimicrobial activity is sparse. In this study, we assessed the pH levels in cutaneous *Leishmania* lesions. *Leishmania major*-infected skin tissue displayed pH levels of 6.7 indicating that lesional pH is acidic. Next, we tested the effect of low extracellular pH on the ability of macrophages to produce leishmanicidal NO and to fight the protozoan parasite *Leishmania major*. Extracellular acidification led to a marked decrease in both NO production and leishmanicidal activity of lipopolysaccharide (LPS) and interferon γ (IFN-γ)-coactivated macrophages. This was not directly caused by a disruption of NOS2 expression, a shortage of reducing equivalents (NAPDH) or substrate (L-arginine), but by a direct, pH-mediated inhibition of NOS2 enzyme activity. Normalization of intracellular pH significantly increased NO production and antiparasitic activity of macrophages even in an acidic microenvironment. Overall, these findings indicate that low local tissue pH can curtail NO production and leishmanicidal activity of macrophages.

## Introduction

Immune responses in infected tissues are not only driven by inflammatory cytokines and mediators, but also by local ionic composition [reviewed in: (1)], metabolism (2–5), and oxygen availability [reviewed in: (6–8)]. Hypoxia is a hallmark of infected tissue [reviewed in: (6–9)]. It triggers anaerobic glycolysis which ultimately contributes to lactic acid production [reviewed in: (10, 11)]. Moreover, infection and inflammation can trigger excess metabolic breakdown of glucose to pyruvate, which surpasses the cell’s capability to fuel it into the mitochondrial respiration [reviewed in: (11, 12)]. Both factors ultimately contribute to accumulation of lactic acid and, thus, induce tissue acidosis [reviewed in: (10, 13)] and in case of systemic infection (sepsis), lactic acidosis [reviewed in: (11)].

The role of lactic acidosis especially in the diagnosis and treatment of septic patients has been studied intensively (reviewed in: [11, 14)]. The influence of local tissue pH, however, on antimicrobial immunity has received less attention. Therefore, to assess the role of an acidic microenvironment, we used a mouse model of cutaneous leishmaniasis, which is induced by the protozoan parasite *Leishmania* (*L.*) *major* (15–17). Control of *L. major* in this model critically depends on the ability of macrophages to produce high levels of leishmanicidal nitric oxide [NO; reviewed in: (18–20)]. The production of NO during cutaneous *L. major* infection not only ensures direct killing of the protozoan parasite [reviewed in: (21–23)], but NO also curtails the parasite’s metabolic activity (24) Moreover, NO impairs the recruitment of monocyte-derived phagocytes to the infectious lesions (25). This mechanism significantly contributes to antimicrobial control as recruited monocyte-derived phagocytes provide an important cellular niche that favor *Leishmania* replication (25–27).

Low extracellular pH levels can reportedly inhibit the activity of the enzyme NOS2 (28–30), which is required for NO production in macrophages. Therefore, we set out to quantify the pH levels in infected cutaneous *Leishmania* lesions and to assess the role of extracellular acidification on the ability of macrophages to fight intracellular *Leishmania*.

## Materials and Methods

### Reagents and Antibodies

Lipopolysaccharide (LPS) from *Escherichia coli* O111:B4, lactic acid (LA), sodium lactate (NaL), L-arginine hydrochloride (Arg-HCl), and L-arginine methyl ester dihydrochloride (Arg-ME) were purchased from Sigma Aldrich (Taufkirchen, Germany), whereas interferon γ (IFN-γ) and 1-[N-(2-aminoethyl)-N-(2-aminoethyl)amino] diazen-1-ium-1,2-iolate (DETA-NO) were obtained from Invitrogen (Darmstadt, Germany) and Cayman Chemical (Ann Harbor, MI), respectively. Hydrochloric acid (HCl) was purchased from Fisher Chemical (Schwerte, Germany). RPMI 1640, DMEM and PBS were purchased from Gibco (Darmstadt, Germany). Immunoblotting was carried out using the following antibodies: rabbit anti-Actin (A2066; Sigma Aldrich), mouse anti-HSP90α/β (sc-7947; Santa Cruz Biotechnology, Heidelberg, Germany), rabbit anti-NOS2 (ADI-KAS-NO001; Enzo Life Sciences, Lörrach, Germany) and mouse anti-Arginase 1 (sc-166920; Santa Cruz). Either swine anti-rabbit HRP (P0399, Dako, Hamburg, Germany) or goat anti-mouse HRP (P0447, Agilent) were used as secondary antibodies.

### Cultivation of *L. major*

*L. major* promastigote strain MHOM/IL/81/FEBNI was propagated in RPMI 1640 (10% fetal calf serum) on Novy-MacNeal-Nicolle blood agar slants for a maximum of five passages and used as described earlier (31, 32). *L. major* promastigotes were collected from blood agar slants. After washing with phosphate-buffered saline (PBS), 3 x 10^6^ parasites were used for infection. For *in vitro* infection of bone marrow-derived macrophages (BMDM), *L. major* were propagated in Schneider’s insect medium (Sigma Aldrich) for a maximum of five passages, washed with PBS and resuspended in RPMI microscopically 1640 complete medium.

### *In Vitro* Infection of Macrophages

BMDM were generated from wildtype C57BL/6NCrl (Charles River Breeding Laboratories, Sulzfeld, Germany) as described earlier (33). Briefly, BMDM were harvested from Teflon bags (FT FEP 100 C; Dupont; purchased *via* APSOparts, Fellbach, Germany) and infected with *L. major* promastigotes with a multiplicity of infection of 30 for 4 h in RPMI 1640 medium, as described earlier (32). Thereafter, cells were washed with PBS and *Leishmania*-infected BMDM were costimulated with 20 ng/mL LPS/IFN-γ each (unless indicated otherwise) in the presence or absence of 10 mM LA in RPMI 1640 medium for indicated period of time. After 72 h, infected BMDM were stained with Diff-Quik (Eberhard Lehmann, Berlin, Germany) and analyzed for determination of the percentage of infected cells. Per high power field up to 34 cells were counted.

### Immunoblotting

Preparation of cell lysates, extraction of proteins, and immunoblotting were carried out as described earlier (32, 34). Proteins were separated on TRIS-glycine gels (7.5% for NOS2 and 12% for Arginase 1) and transferred to PVDF membranes. Staining with appropriate primary and secondary antibodies was followed by signal visualization using the Chemo Star Imager (Intas Science Imaging Instruments, Göttingen, Germany). Densitometry of signals was done using ImageJ (Version 1.52a; Rasband, W., ImageJ, National Institutes of Health, USA, https://imagej.nih.gov/ij/).

### Nitrite Production

Accumulation of nitrite in cell supernatants was quantified by the Griess reaction, as described earlier (32). In brief, supernatants of infected and/or stimulated BMDM were mixed with equal amounts of Griess reagent 1 (1% sulfonamide in 5% phosphorous acid) and Griess reagent 2 (0.1% N-1-naphtyletylenediamine in H_2_O). Sodium nitrite (Sigma) was used as standard. Absorbance was recorded at 540 nm using an iMark™ microplate absorbance reader (Bio-Rad, Feldkirchen, Germany).

### Gene Expression Analysis

As described earlier (32), total RNA was extracted from stimulated cells after 24 h with TriFAST reagent (VWR International, Ismaning, Germany) and subjected to reverse transcription (high-capacity cDNA reverse transcription kit, Applied Biosystems, Darmstadt, Germany). Quantitative real-time PCR was performed on ABI Prism 7900 sequence detector (Applied Biosystems) using FastSTart Universal Probe Master (Rox) (Roche Diagnostics, Mannheim, Germany) and the following TaqMan probes: Hypoxanthine phosphoribosyltransferase 1 (*Hprt1*; Mm03024075_m1), *Nos2* (Mm00440502_m1), and *Arg1* (Mm00475988_m1). These probes were purchased from Applied Biosystems. The ΔΔcCT method was used for quantification. The ratio of target mRNA to control *Hprt1* in non-stimulated (ns) specimen was set to 1.

### NADPH/NADP^+^ Quantification

NADPH/NADP^+^-ratio was determined according to manufacturer’s instruction using the NADP/NADPH-Glo™ Assay (Promega GmbH, Walldorf, Germany) which allows for quantification of NADP/NADPH using a single-reagent. Briefly, BMDM were costimulated with LPS/IFNγ in absence or presence of lactic acid for 24 h and then mixed with NADP/NADPH-Glo™ reagent which quantifies NADP/NADPH in a single-step. Cells were incubated for 30 min at room temperature and luminescence was recorded on a Viktor3™ multilabel plate reader (PerkinElmer, Rodgau, Germany).

### Arginine Quantification

BMDM were washed three times with PBS to remove remaining traces of cell culture medium. Then, 80% of cold aqueous methanol was added and samples were immediately frozen at −80°C. As described earlier for amino acid analysis (35), samples were thawed and 10 µL of an internal standard mix containing uniformly ^13^C- and ^15^N-labeled amino acids (MSK-CAA-1, Euriso-Top GmbH, Saarbrücken, Germany) and deuterated ornithine and deuterated hippuric acid were added. The samples were vortexed, and centrifuged at 9,560 x g for 5 min at 4°C. The supernatants were collected, followed by addition of 200 µL 80% methanol, vortexing and centrifugation to wash the pellets. The wash steps were repeated, but the samples were centrifuged at a higher speed (13,800 g). All supernatants were combined and dried in a vacuum evaporator (CombiDancer, Hettich AG, Bach, Switzerland) followed by reconstitution in 100 μL pure water. Amino acid analysis by HPLC-ESI-MS/MS (HPLC 1200 (Agilent, Waldbronn, Germany) with API 4000 QTRAP (AB SCIEX, Darmstadt, Germany) or ExionLC AD with Triple Quad 6500+, AB SCIEX) after derivatization with propyl chloroformate/propanol was performed using a 10 µL aliquot of the sample extract as described (36).

Intracellular amino acid amounts were normalized to total protein content, which was determined using an assay based on the fluorescent dye SERVA Purple (Serva, Heidelberg, Germany) as recently described (37). The relative arginine content was calculated in relation to the mean value of the LPS/IFN-γ costimulated cells of the respective experiment.

### Recombinant NOS2 Enzyme Activity

For determination of recombinant NOS2 enzyme activity, 2 units of recombinant enzyme (Biomol, Hamburg, Germany) were incubated in appropriate buffer following the manufacturer’s protocol. In short, 50 mM HEPES (Carl Roth, Karlsruhe, Germany) and 1 mM magnesium acetate (Sigma Aldrich) were adjusted to pH 7.4 or 6.0 by addition of hydrochloric acid (Carl Roth). Prior to buffer use, 0.15 mM NADPH (Sigma Aldrich), 4.5 µM oxyhemoglobin (Sigma Aldrich), 18 µM tetrahydrobiopterin (Sigma Aldrich), and 180 µM dithiothreitol (Sigma Aldrich) were freshly added. 1 mM Arg-HCl (Sigma Aldrich) was also freshly added where indicated. After incubation at 37°C, nitrite accumulation was quantified at distinct time points between 1 h and 8.5 h using the Griess assay (32).

### Measurement of Extracellular pH Levels *In Vitro*

pH levels in culture medium were measured non-invasively by using the PreSens technology (PreSens Precision Sensing GmbH, Regensburg, Germany), as described earlier (38). 0.5x10^6^ BMDM were seeded in 24-well Hydrodish HD24 plates in 1 mL RPMI 1640 medium under cell culture conditions for the indicated period of time. pH levels were continuously monitored using the SDR SensorDish^®^ Reader. Data were analyzed with SDR_v38 software package (Presens Precision Sensing GmbH, Regensburg, Germany).

### Quantification of Intracellular pH in BMDM

Essentially, quantification of intracellular pH in BMDM followed an earlier published protocol (39). BMDM were incubated with indicated treatments for 24 h in RPMI 1640 medium. Afterwards, cells were loaded with 10 µM carboxy SNARF-1 AM acetate (Thermo Fisher Scientific, #C1272) in 1 mL Hank’s balanced salt solution (HBSS, containing 2 g/L NaHCO3) for 30 min and subsequently incubated with or without 10 mM lactic acid for 10 min in the presence or absence of increasing concentrations of Arg-HCl. For calibration curves, an intracellular pH calibration buffer kit (Thermo Fisher Scientific, #P35379) was used. In brief, BMDM were incubated with a mix of pH-controlled buffers and valinomycin/nigericin according to the manufacturer’s instructions. Intracellular pH was assessed by flow cytometry. In detail, pH-dependent spectral shifts of SNARF-1 were recorded and the ratios of the emission wavelength at λ1, transmitted by a 585/42 BP filter, to the wavelength at λ2, transmitted by a 670 LP filter were calculated, as described earlier (39). Treatment induced changes were delineated from the calibration curve, performed for each experiment. FlowJo software version 10.7.1 was used for data analysis.

### Measurement of Lesional pH

All animal experiments followed a protocol that had been approved by the Animal Welfare Committee of the local governmental authority (Regierung von Unterfranken, Würzburg, Germany). Mice were infected subcutaneously with 3 x 10^6^ stationary-phase *L. major* promastigotes (of low *in vitro* passage [≤ 5] in the right hind footpad in 50 µL PBS), as described earlier (32). 14 days post infection, mice were sacrificed and, immediately thereafter, footpad pH was determined by a micro fiber optic pH meter with needle-type housed pH microsensors (20/0.4) using a manual micromanipulator (PreSens Precision Sensing GmbH) essentially as described earlier (38). After overnight calibration according to the manufacturer’s protocol, the microsensor was inserted 3 - 4 mm into the footpad and pH values were recorded over a period of 1 - 3 min after insertion of the pH sensor with the software pH1-View (PreSens Precision Sensing GmbH).

### Statistical Analysis

Data is expressed as mean ± SEM (unless indicated otherwise). Statistical analysis was carried out using Prism v6.0 or v8.0 software (GraphPad). Outliers were identified using the ROUT test (**Figures 4A, D**). Comparing two groups, unpaired Student’s t*-test* ± Welch correction (if unequal variances were detected by the F-Test) was applied for datasets where the Kolmogorov-Smirnow test indicated normal distribution. Otherwise, the Mann-Whitney test was used. Multiple groups were tested either with Kruskal-Wallis test in combination with Dunn multiple-comparison test for not normally distributed data points or ANOVA followed by Bonferroni’s multiple-comparison test for normally distributed data. Unless indicated otherwise, *p*-values <0.05 were considered as significant.

## Results

### Leishmanial Skin Lesions Are Acidic

**Figure 1:**
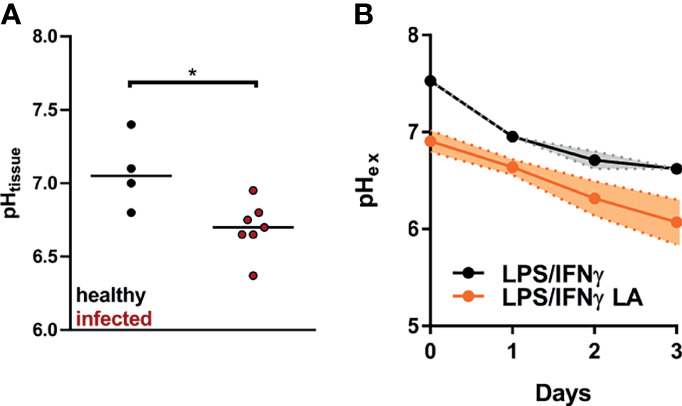
pH levels found in *L. major* infected lesions can be simulated by addition of 10 mM lactic acid to LPS/IFN-γ-coactivated macrophages. **(A)** C57BL/6 mice were infected with *L. major* in their hind footpads. At day 14 post infection tissue pH (pH_tissue_) was determined in healthy and infected tissue (median, n = 4-7 animals from two independent experiments). *p < 0.05, Mann-Whitney test. **(B)** BMDM were stimulated with LPS (10 ng/mL)/IFN-γ (20 ng/mL) for 24 h. Where indicated, cells were exposed to 10 mM lactic acid (LA). Determination of extracellular pH (pH_ex_; mean + SEM, n = 3-5 from three independent experiments).

In our experimental setup, C57BL/6 mice usually develop a clear clinical lesion at day 14 after infection. At this point, the cutaneous lesion has reached its maximum or it barely enlarges thereafter, before it heals over several weeks (32, 40). Lesional pH was determined 14 days after infection with *L. major* in cutaneous lesions and contralateral uninfected healthy skin tissue. The pH of uninfected tissue was 7.1, while the pH of *Leishmania-*infected lesions dropped to 6.7, indicating that lesional pH is acidic (**Figure 1A**).

*In vitro*, addition of 10 mM lactic acid to LPS/IFN-γ-costimulated macrophages resulted in an immediate steep decrease in extracellular pH. Compared with LPS/IFN-γ-costimulated macrophages, pH remained lower in cells additionally treated with lactic acid throughout the 72 hour observation period. At 24 hours after the addition of lactic acid, this simulated quite well the situation *in vivo.* (**Figure 1B**).

### Extracellular Acidification Impairs NO Production and Leishmanicidal Macrophage Activity

**Figure 2:**
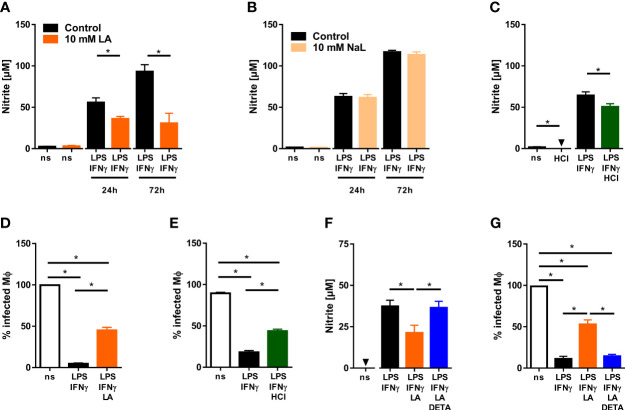
Low pH reduces NO production and impairs anti-leishmanial defenses of LPS/IFN-γ-coactivated macrophages. **(A, B)** BMDM were stimulated with LPS (10 ng/mL)/IFN-γ (20 ng/mL) or left unstimulated (ns) for 24 - 72 h. Where indicated, 10 mM lactic acid (LA) or 10 mM sodium lactate (NaL) were added. **(A)** Nitrite levels were determined at indicated time points (means + SEM, n = 10-29 samples from at least four independent experiments; *p < 0.01, Student’s t-test + Welch correction or Mann-Whitney test). **(B)** Nitrite levels were determined at indicated time points (mean + SEM, n = 16-40 samples from ten independent experiments; *p <0.01, Mann-Whitney test). **(C)** As in **(A)**, but cells were stimulated with 10 mM HCl for 24 h (means + SEM; n = 12-16 from at least two independent experiments; *p < 0.05, Student’s t-test or Mann-Whitney test). **(D–G)** BMDM were infected with *L. major* and costimulated with LPS (20ng/mL)/IFN-γ (20 ng/mL) or left unstimulated (ns) for 72 h. Where indicated cells were additionally exposed to 10 mM HCl or 10 mM LA and/or 25 µM DETA-NO (DETA). **(D)** Infection rate (mean + SEM, n = 29-30 high power fields from three independent experiments; *p < 0.05, Kruskal-Wallis test and Dunn *post hoc* test). **(E)** Infection rate (mean + SEM, n = 16 high power fields from two independent experiments; *p < 0.05, ANOVA with Bonferroni’s test). **(F)** Nitrite content of supernatants (mean + SEM, n = 14 from three independent experiments; *p < 0.05, Kruskal-Wallis test and Dunn *post hoc* test. **(G)** Infection rate (mean + SEM, n = 18 high power fields from three independent experiments; *p < 0.05, Kruskal-Wallis test and Dunn *post hoc* test).

Macrophages play a critical role in fighting *Leishmania* [reviewed in: (20, 41)]. Therefore, we tested whether acidification of the extracellular microenvironment would influence the ability of LPS/IFN-γ-cotreated macrophages to produce leishmanicidal NO and to ward off *Leishmania*. Addition of 10 mM lactic acid to LPS/IFN-γ-costimulated macrophages curtailed their ability to produce NO (**Figure 2A**). Its sodium salt [sodium lactate (NaL)], in contrast, did not impact the release of NO from macrophages (**Figure 2B**). Extracellular acidification with hydrochloric acid (HCl) also impaired NO production of LPS/IFN-γ-coactivated macrophages (**Figure 2C**). Decreased NO release under acidic conditions was accompanied by a reduced ability of the macrophages to clear *Leishmania* (**Figures 2D, E**). Addition of NO donor 1-[N-(2-aminoethyl)-N-(2-aminoethyl)amino] diazen-1-ium-1,2-iolate (DETA-NO) restored not only NO levels (**Figure 2F**) but also leishmanicidal activity (**Figure 2G**) of activated macrophages exposed to 10 mM lactic acid.

### Induction of *Nos2* mRNA and Protein Expression Is Maintained Upon Exposure to Lactic Acid

**Figure 3:**
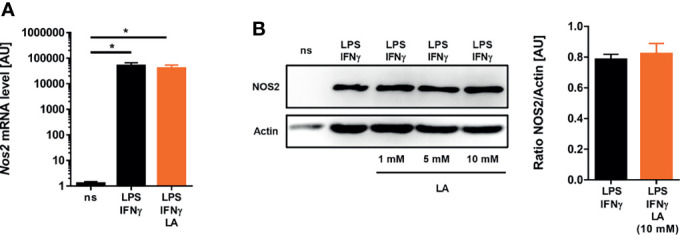
Induction of *Nos2* on mRNA and protein level is preserved upon exposure to lactic acid in LPS/IFN-γ-coactivated macrophages. **(A, B)** BMDM were costimulated with LPS (10 ng/mL)/IFN-γ (20 ng/mL) or left unstimulated (ns) for 24 h. Cells were treated with 10 mM lactic acid (LA), unless indicated otherwise. **(A)** *Nos2* mRNA levels (mean + SEM, n = 14-15 samples from five independent experiments; *p < 0.05, Kruskal-Wallis test and Dunn *post hoc* test). **(B)** Left panel: NOS2 and Actin protein levels (representative out of six similar independent experiments). Right panel: Densitometry of NOS2 normalized to Actin protein levels after treatment with 10 mM LA (mean + SEM, n = 6 from six independent experiments; Student’s *t*-test).

Next, we wanted to elucidate the mechanism that underlies the reduced ability of macrophages to produce NO. Production of high-level NO in macrophages hinges on the ability of macrophages to induce the expression of the type 2 NO synthase (NOS2) [reviewed in: (19, 20)]. In contrast to only LPS-stimulated macrophages (28, 30), addition of lactic acid to LPS/IFN-γ-costimulated macrophages did not interfere with *Nos2* expression at both the mRNA (**Figure 3A**) and protein (**Figure 3B**) level. From these findings we concluded that reduced NO production was not caused by impaired *Nos2* expression.

### NADPH- and L-Arginine Availability Do Not Explain Reduced NO Production of Acid-Exposed Macrophages

**Figure 4:**
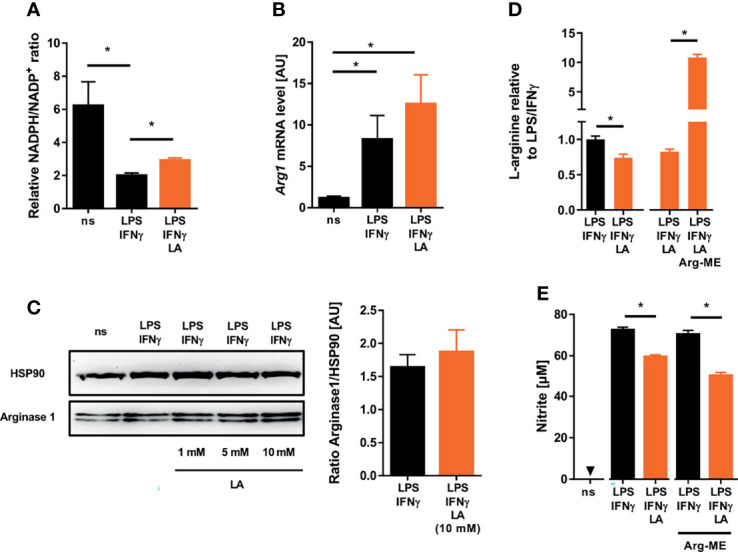
Lactic acid-induced changes in cosubstrate availability do not underlie impaired NO production in LPS/IFN-γ coactivated macrophages. **(A–E)** BMDM were costimulated with LPS (10 ng/mL)/IFN-γ (20 ng/mL) or left untreated (ns) for 24 h. Cells were treated with 10 mM lactic acid (LA), unless indicated otherwise. **(A)** Relative NADPH/NADP^+^-ratio (mean + SEM, n = 11-12 from three independent experiments; *p < 0.05, Kruskal-Wallis test and Dunn *post hoc* test). **(B)** *Arg1* mRNA levels (mean + SEM, n = 14-15 from five independent experiments; *p < 0.05, Kruskal-Wallis test and the Dunn *post hoc* test). **(C)** Left panel: Arginase 1 and HSP90 protein levels (representative of six similar independent experiments). Right panel: Densitometry of Arginase 1 normalized to HSP90 protein levels after treatment with 10 mM LA (mean + SEM, n = 6 from six independent experiments; Mann-Whitney test). **(D)** Relative L-arginine levels (mean + SEM, n = 9-28 from at least two independent experiments; *p < 0.05, Student’s *t*-test or Mann-Whitney test). **(E)** LPS/IFN-γ stimulated BMDM ± LA were treated with 10 mM L-arginine methyl ester dihydrochloride (Arg-ME) for 24 h. Nitrite accumulation in supernatants (mean + SEM, n = 16 from two independent experiments; *p < 0.05, Mann-Whitney test).

Alternatively, addition of lactic acid might affect the availability of L-arginine and NADPH, both of which are required for NO production by NOS2 [reviewed in: (42)]. Extracellular acidification increased the NADPH/NADP^+^-ratio compared to controls suggesting that reduced NO production is not due to lack of NADPH (**Figure 4A**). Acidic conditions can induce the expression of arginases (43) and, thereby, limit the availability of L-arginine. Here, addition of lactic acid to LPS/IFN-γ-costimulated macrophages led to a minor, non-significant increase in Arginase 1 (Arg1) expression at both the mRNA (**Figure 4B**) and protein levels (**Figure 4C**), which nonetheless resulted in a significantly reduced intracellular pool of L-arginine (**Figure 4D**). Next, we tested the impact of L-arginine shortage on NO release from activated macrophages exposed to acidic conditions. For that purpose, we used cell permeable L-arginine methyl ester dihydrochloride (Arg-ME) to replenish the intracellular L-arginine pool (**Figure 4D**). In contrast to Nω-nitro-L-arginin**e** methyl ester hydrochloride [reviewed in: (44)], Arg-ME did not inhibit NO production (**Figure 4E**). Of note, addition of Arg-ME to infected macrophages did not restore NO production by LPS/IFN-γ-costimulated macrophages exposed to acidic conditions (**Figure 4E**). This suggests that the reduced L-arginine availability is not limiting NO production under acidic conditions.

### Low pH Levels Directly Inhibit Enzymatic NOS2 Activity

**Figure 5:**
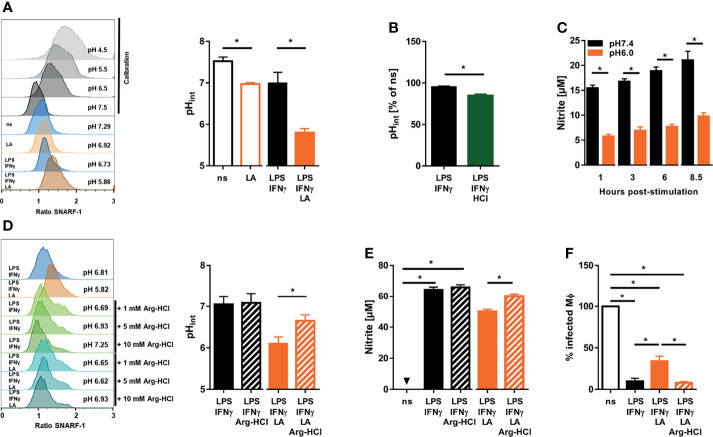
Exposure to lactic acid triggers low intracellular pH levels which directly impair NOS2 enzyme activity in LPS/IFN-γ coactivated macrophages **(A)** BMDM were costimulated with LPS (10 ng/mL)/IFN-γ (20 ng/mL) or left unstimulated (ns) for 24 h. Where indicated, cells were treated with 10 mM lactic acid (LA). Determination of intracellular pH. Left panel: representative histograms out of four similar independent experiments upon exposure to calibration buffers (in grey), unstimulated (ns), 10 mM lactic acid (LA), and LPS/IFN-γ ± LA treated BMDM. Right panel: intracellular pH (mean + SEM, n = 4 samples from four independent experiments; *p < 0.05, Mann-Whitney test. **(B)** BMDM were costimulated with LPS (10 ng/mL)/IFN-γ (20 ng/mL) for 24 h. Where indicated, cells were treated with additional 10 mM hydrochloric acid (HCl). Determination of intracellular pH in percent of unstimulated (ns) cells (mean + SEM, n = 4 samples from four experiments; *p < 0.05, Mann-Whitney test). **(C)** *In vitro* activity of recombinant NOS2 at different pH values over time. Nitrite accumulation (mean + SEM, n = 9 from four independent experiments; *p < 0.05, Student’s *t*-test or Mann-Whitney test). **(D)** As in **(A)**, but 1, 5 or 10 mM L-arginine monohydrochloride (Arg-HCl) were added where indicated. Left panel: histogram of LPS/IFN-γ ± LA stimulated BMDM cotreated with Arg-HCl (representative out of five similar independent experiments). Right panel: intracellular pH of LPS/IFN-γ ± LA stimulated BMDM exposed to 10 mM Arg-HCl (mean + SEM, n = 5 samples from five independent experiments; *p < 0.05, Student’s *t*-test). **(E)** As in **(A)**, but 10 mM Arg-HCl was added, where indicated. Nitrite accumulation (mean + SEM, n = 29-38 from seven independent experiments; *p < 0.05, Kruskal-Wallis test and Dunn *post hoc* test or Mann-Whitney test). **(F)** BMDM were infected with *L. major* and costimulated with LPS (20 ng/mL)/IFN-γ (20 ng/mL) ± 10 mM Arg-HCl for 72 h. Infection rate (mean + SEM, n = 26 high power fields from five independent experiments; *p < 0.05, Kruskal-Wallis test and Dunn *post hoc* test).

Extracellular acidification can trigger intracellular acidification (45). Therefore, we used the pH-sensitive dye SNARF-1 to monitor intracellular pH levels (46). In line with earlier findings in mouse dendritic cells (47), we found that coactivation of macrophages with LPS/IFN-γ resulted in a drop in intracellular pH (**Figure 5A**). More importantly, exposure of LPS/IFN-γ costimulated macrophages to acidic conditions resulted in a further significant decrease in intracellular pH (**Figures 5A, B**). Acidic pH is known to affect the activity of various enzymes [reviewed in: (48)] including NOS2 (29). Therefore, we tested whether the pH (pH 5.81 ± 0.19) encountered within LPS/IFN-γ-costimulated macrophages upon exposure to lactic acid (**Figure 5A**) was able to inhibit the enzymatic activity of recombinant NOS2 directly. These experiments showed that enzymatic NOS2 activity at pH 6.0 was substantially diminished compared to its activity at pH 7.4 (**Figure 5C**).

### Increasing Intracellular pH Restores Leishmanicidal Activity of Macrophages in an Acidic Microenvironment

Infusion of arginine hydrochloride (Arg-HCl) lowers extracellular pH in rats, but increases intracellular pH levels (49). In line with this, we found that Arg-HCl treatment significantly elevated the intracellular pH levels in LA-exposed macrophages *in vitro* (**Figure 5D**). This was accompanied by a significant increase in NO production (**Figure 5E**) and leishmanicidal activity (**Figure 5F**) of LPS/IFN-γ-cotreated macrophages exposed to lactic acid. Taken together, these findings demonstrate that normalization of intracellular pH is able to restore NO production and leishmanicidal macrophage activity in acidic microenvironments.

## Discussion

In this report, we provide evidence that extracellular acidification resulted in direct inhibition of NOS2 enzyme activity and subsequent reduced production of leishmanicidal NO in macrophages, which ultimately impeded their antimicrobial activity. In addition to cancerous tissues [reviewed in: (50, 51)], increased proton concentrations and tissue acidosis are found in inflamed, infected, and ischemic tissues [reviewed in: (10, 13, 52)]. We reported earlier that *Leishmania* skin lesions displayed low oxygen levels when the skin lesions reached their maximum size around 14 days post infection (40). Our findings of low lesional tissue pH at day 14 after infection conforms with this, because low tissue oxygenation is paralleled by increased proton levels in afflicted tissues [reviewed in: (10, 11)]. Healing of *Leishmania* lesions was accompanied by normalization of tissue oxygenation (40). It is tempting to speculate that resolution of disease is associated with restoration of lesional pH to normal as well. The mechanisms that trigger low tissue pH warrant further investigation. For instance, following and extending earlier reasoning [reviewed in: (10)], it is possible that infection-associated cell death [reviewed in: (53)], infection-induced increases in glycolysis and metabolic reprogramming [reviewed in: (12)] and/or infection-triggered induction of low tissue oxygen [reviewed in: (6)] contribute to low tissue pH.

Increases in extracellular proton concentrations are able to impact the function of immune cells [reviewed in: (54, 55)]. For instance, acidic microenvironments are able to activate immature dendritic cells (56) and to promote the phagocytic capacity of neutrophils (57). Moreover, acidosis can trigger inflammasome activation in human macrophages (58). Acidic conditions can increase the production of interleukin-1β by murine macrophages in response to *Pseudomonas aeruginosa* infection (59). Based on these findings, extracellular acidosis has been proposed as a “danger signal” (58).

In tumor environments, low pH potentiates the immunosuppressive function of macrophages and thereby promotes tumor growth (43, 60). Although many antimicrobial peptides require low pH for their optimal activity [reviewed in: (61)], to the best of our knowledge, data on the impact of low pH on antimicrobial macrophage activity is limited. Extracellular acidosis reportedly enhances Zika virus replication in various cells including human monocytes (62). Enhanced viral replication in this study was linked to increased viral attachment to heparan sulphate, which is expressed on the surface of host cells (62). In a porcine model of cystic fibrosis, airway surface liquid was more acidic compared to healthy lungs (63). This pH reduction in the airway surface liquid inhibited the antimicrobial activity (63). Interestingly, the airway mucosal microenvironment undergoes large excursions in pH during breathing (64). Transient alkalinization seems to be important for host defense (64).

Here, we demonstrate that increases in extracellular protons curtail LPS/IFN-γ-triggered NO production, which is critical for anti-leishmanial control in macrophages (21–23). Our findings are in line with others who have found decreased NO production in macrophages (28) and mesangial cells (29) cultivated under acidic conditions. However, the opposite observation of increased NO production by macrophages upon exposure to increased extracellular proton availability has also been reported (30, 65). A comprehensive model that reconciles these disparate findings into a unified concept does not exist yet. Differences in the experimental setup might explain the divergent findings. For instance, using HCl to adjust pH, exposure of the LPS-stimulated mouse macrophage-like cell line RAW 264.7 to mild acidic conditions increased NO release, while harsh acidic conditions resulted in suppressed NO production (30). Of note, in the same study lactic acid inhibited NO production in a dose dependent manner (30). In this situation, reduced NO production upon exposure to acidic conditions was correlated with impaired binding of the nuclear factor (NF)-κB to DNA and reduced *Nos2* expression (30). In line with this, acidic conditions are able to impair *Nos2* gene expression in renal kidney cells directly (66). When we compared cells costimulated with LPS and IFN-γ and tested the impact of increased proton concentrations on the expression of *Nos2*, we did not detect a significant difference. This suggests that in our experimental setup transcriptional regulation of *Nos2* was not linked to a decrease in NO production. It is possible that costimulation with LPS and IFN-γ overrode the effect of low pH on NF-κB-dependent transcriptional responses in cells which were only stimulated with LPS.

Further, our experimental data does not suggest that the sole lack of L-arginine and/or NADPH/H^+^ caused the observed decrease in NO production by LPS/IFN-γ-costimulated macrophages exposed to lactic acid. Rather, intracellular acidification impairs enzymatic activity of NOS2 directly. This notion is supported by at least partial rescue of both, NO production and leishmanicidal activity in macrophages upon treatment with arginine hydrochloride, which was paralleled by a partial normalization of intracellular pH. This very much recapitulates earlier findings in mesangial cells, where low pH levels directly interfered with NOS2 enzyme activity (29). Intracellular acidification has also been shown to impair superoxide and hydrogen peroxide production of neutrophils, which could further diminish antimicrobial control and support microbial proliferation (67).

Since administration of bicarbonate promoted cancer immunotherapy (68, 69), buffering might represent a strategy to reduce the suppressive effects of acidification on immune cell activation and, ultimately, on their antimicrobial potential. In the case of *Leishmania* infection, it is tempting to speculate that buffering might increase local NO production in leishmanial lesions. This could enhance direct leishmanicidal activity (22) and, in addition, might inhibit the excess influx of mononuclear cells, which in turn serve as cellular niches for *Leishmania* replication (25). Whether bicarbonate and/or Arg-HCl treatment are useful for this purpose will require additional experimentation, which, for instance, may include monitoring of lesional pH, tissue NO levels and parasite burden.

In addition, the mechanisms that facilitate entry of protons into macrophages that ultimately result in intracellular acidosis are unclear and warrant further investigation. For instance, the transient receptor potential cation channel subfamily V member (TRPV) 1 is able to sense increased availability of protons and to increase proton concentrations in neurons (70). TRPV1 is also expressed on macrophages (71). Moreover, other transporters, channels, and exchangers such as Na^+^/H^+^ exchangers or monocarboxylate transporters (39, 47, 72–75) may play a role as well. Finally, the cellular mechanism that results in at least partial normalization of intracellular pH by addition of Arg-HCl is unclear and warrants further investigation.

In summary, our findings demonstrate that leishmanial lesions displayed low pH and that acidic conditions impaired the leishmanicidal activity of macrophages *via* inhibition of NOS2 enzyme activity. Of note, normalization of the intracellular pH largely restored NO production and leishmanicidal activity even in an acidic microenvironment.

## Data Availability Statement

The raw data supporting the conclusions of this article will be made available by the authors, without undue reservation.

## Ethics Statement

The animal study was reviewed and approved by Animal Welfare Committee of the local governmental authority (Regierung von Unterfranken Würzburg, Germany).

## Author Contributions

LF, LH, KR, MV, NB, AW, SW, SH, and VS acquired, analyzed and interpreted data. JG, PO, RB, KD, and KR interpreted data and contributed to the design of experiments. LF, LH, VS, MK, and JJ interpreted data and designed and conceptualized experiments. JJ and MK oversaw the study. JJ, VS, and MK provided the first draft of the manuscript. All authors read and approved the final version of the manuscript.

## Conflict of Interest

The authors declare that the research was conducted in the absence of any commercial or financial relationships that could be construed as a potential conflict of interest.

## Publisher’s Note

All claims expressed in this article are solely those of the authors and do not necessarily represent those of their affiliated organizations, or those of the publisher, the editors and the reviewers. Any product that may be evaluated in this article, or claim that may be made by its manufacturer, is not guaranteed or endorsed by the publisher.
